# Innovative CO_2_-NBs-assisted ultrasonication for the phytochemical extraction of peanut (*Arachis hypoga*) shells: Synthesis and characterization of CO_2_-nanobubbles

**DOI:** 10.1016/j.ultsonch.2024.107198

**Published:** 2024-12-12

**Authors:** Nayyar Iqbal, Miral Javed, Ramy M. Khoder, Areej Areej, Renyu Zheng, Shanbai Xiong, Ibrahim Khalifa, Hassan Barakat, Youming Liu

**Affiliations:** a College of Food Science and Technology/National R&D Branch Center for Conventional Freshwater Fish Processing (Wuhan), Huazhong Agricultural University, Wuhan 430070, PR. China; b School of Food Science and Biological Engineering, Jiangsu University, Zhenjiang, China; c Zhejiang University Hangzhou, Zhejiang Province, 310000, China; d Food Technology Department, Faculty of Agriculture, Benha University, Moshtohor, Toukh 13736, Egypt; e Department of Food Science and Human Nutrition, College of Agriculture and Food, Qassim University, Buraydah 51452, Saudi Arabia

**Keywords:** Peanut shell, Polyphenols, CO_2_-Nanobubbles, Ultrasonic extraction, HPLC, SEM

## Abstract

This study was designed to obtain the maximum extraction yield of peanut shell (PS) polyphenols using a novel carbon dioxide nanobubbles (CO_2_-NBs) assisted ultrasonic extraction method. CO_2_-NBs were generated in distilled water with a self-developed high-pressure nano-jet homogenization method and characterized by size, zeta potential and transmission electron microscopy (TEM). The obtained nanobubble’s mean size and zeta potential were 229.96 ± 17.44 nm and −15.9 ± 1.27 mV, respectively. Later, these CO_2_-NBs, combined with ultrasonic method, were used for the extraction of polyphenols, achieving the highest polyphenol content (3619.21 ± 113.07 µg GAE/mL) as compared to ultrasonic extraction (2914.69 ± 145.45 µg GAE/mL) and conventional extraction (2340.11 ± 80.02 µg GAE/mL). Response surface methodology (RSM) provided optimization parameters, including ultrasonic power of 358.76 W, surfactant concentration of 4.54 %, and extraction time of 41.41 min. HPLC analysis identified distinct peaks corresponding to polyphenolic compounds such as gallo-catechin, catechin gallate, resveratrol, and luteolin, confirming their presence and concentrations in the peanut shell extract (PSE). Scanning electron microscopy (SEM) revealed significant structural disruption and increased porosity in peanut shell powder, supporting the enhanced extraction of polyphenols through CO_2_-NBs-assisted ultrasonic extraction process. This research establishes theoretical and practical foundation for generation of CO_2_-NBs and CO_2_-NBs ultrasonic extraction technology to efficiently extract polyphenols from waste PS, thereby enhancing the extraction efficiency of valuable compounds for use in functional food products and promoting sustainable practices in food industry.

## Graphical abstract

**Figure:**
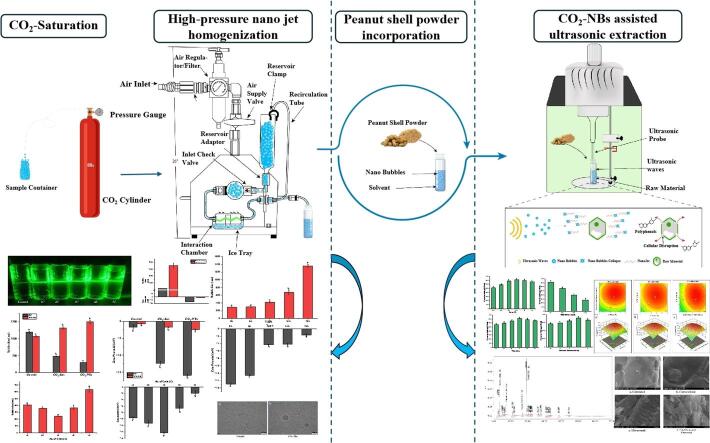

## 1 Introduction

Peanut shells (PS), a by-product of peanut crops, make up around one-third of the weight of the peanut pod and are widely available at low cost [1]. Nevertheless, most PS, being agricultural by-products, were either used as fodder and fuel or left behind, resulting in a significant waste of natural resources. Asia accounts for over 70 % of world peanut production, with China being the major cultivar, producing around 19,000 metric tons for year 2023–2024 [2]. Recent research has shown that PS contain a significant amount of flavonoids and polyphenols, which have the potential to promote good health [3]. PS phytochemicals can be extracted and used as food additives for increased health benefits and good rheological properties.

Extraction is a crucial step for the removal of vital bioactive components to the maximum yield while keeping their functional characteristics. Utilizing novel techniques for bioactive extraction can increase the efficiency of the process and enhance the nutritional and technological benefits of the end product [1]. Among the extraction techniques, Soxhlet extraction using petroleum ether [4], heating, maceration [5], innovative techniques such as microwave-assisted extraction [6], ultrasonic-assisted extraction [7], subcritical fluid extraction [8], pressurized liquid extraction [9] were widely used and discussed in the literature. The approaches adopted were time-consuming, costly, and difficult to implement on a large scale for processing plants. Furthermore, the activity of certain natural antioxidants can occasionally be diminished due to their degradation during the isolation and purification process [10]. Such processes also needed a high cost, high temperature, and significant time [11]. To avoid chemical solvents, high costs, and environmental issues, novel extraction methods are needed to enhance efficiency and sustainability while minimizing energy use and ecological impact.

Emerging research and technology in recent years have focused on nanoscale materials, which have the potential to increase several manufacturing industries, including food, agriculture, polymer, and medical sectors. Nanobubbles are gas bubbles that exist in liquid and have an average diameter of less than 1000 nm [12], possessing characteristics such as a large surface area, great efficiency in transferring mass, and long-term stability [13]. There are numerous applications for nanobubbles, including the treatment of ground waste and drinking water [14], decontamination of sediments and soils along with its uses in biomedical engineering, and other industries including agriculture, fisheries, food [12], and cancer drugs [15]. Over the past ten years, a number of methods have been used to generate NBs, including cavitation, electrolysis, ultrasonication combined with Pd-coated electrodes, and temperature gradients [16], [17], [18], [19], [20]. However, these methods are time-consuming and produce irrational sizes as well as undesirable gasses. Meanwhile, there is an increasing demand for innovative, dependable, and skilled methods that can produce NBs with greater efficiency at a low cost.

Recent studies have demonstrated that ultrasonic-assisted extraction represents an efficient and environmentally sustainable technology that enhances extraction yields while offering advantages of process consistency, minimal solvent consumption, reduced energy input, and shorter processing times compared to conventional methods. The CO_2_-NBs assisted ultrasonic extraction technique, recently developed and used for extracting polyphenols from *Camellia oleifera* shells by Javed et al. [21], is now being explored for the first time to extract polyphenols from PS. This method employs the environmentally friendly properties of CO_2_ and ultrasonic technology to potentially optimize the extraction process for PS, which has not been attempted before.

We hypothesize that high-pressure nano-jet homogenization will create intense cavitation effects leading to the generation of stable CO_2_-NBs, which, when exposed to ultrasonication, will function as nanoscale jets, thereby enhancing the mass transfer and extraction efficiency of polyphenolic compounds from PS. Herein, the purpose of the study is to develop a first-of-its-kind high-pressure nano-jet homogenization technique to prepare the CO_2_—NBs and evaluate a new green and efficient technology to extract phenolic compounds from PS, by employing the CO_2_—NBs-assisted ultrasonic technology. The study seeks to characterize the CO_2_—NBs by measuring size, zeta potential, and morphology by TEM, optimize extraction parameters using RSM, and characterize PSE to validate the system efficiency as compared to the conventional method. This study lays a novel method to develop CO_2_—NBs and provides application potential of sustainable CO_2_—NBs-assisted ultrasonic technology.

## 2 Material and methods

### 2.1 Materials

Peanut shells (PS) were collected from Hubei, China. Ethanol, gallic acid, rutin, sodium nitrate, formic acid, aluminum chloride, sodium carbonate, sodium hydroxide, and FCR reagent were purchased from Sinopharm Chemical Reagent Co., Ltd. (Shanghai, China). ABTS and FRAP assay kits were bought from Beyotime Biotechnology, Shanghai, China. DPPH and all polyphenol standards were purchased from Shanghai Yuanye Bio-Technology Co., Ltd. (Shanghai, China).

PS underwent two rounds of washing using a 1:2 ratio (w/v) of distilled water (dH_2_O). Then, PS was kept in a warm air dryer at 50 °C for 24 h and grounded into powder using a grinder (Midea, BL1206A model-MJ-BL1206A, Guangdong, China). The resulting powder was then passed through a mesh with a pore size of 0.99 mm.

### 2.2 Generation of CO_2_-NBs

**Fig. 1:**
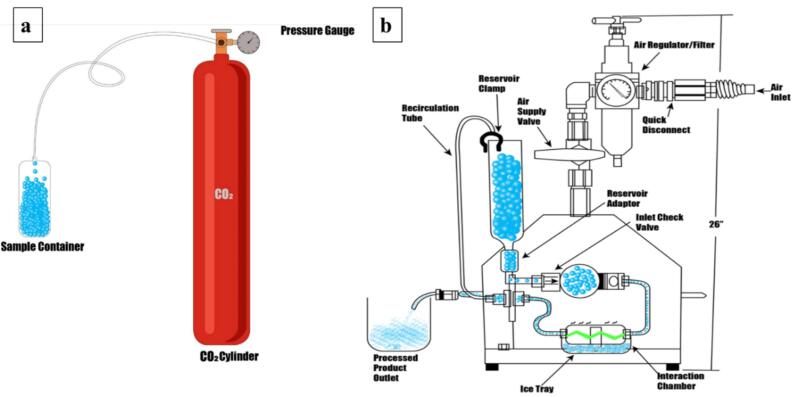
Generation method of CO_2_-Nanobbules, (a) Saturation of CO_2_ gas in a solvent, (b) Generation of CO_2_-NBs by high-pressure nano-jet homogenizer.

Nano-bubbles were generated with a novel self-developed method. Two different solvents, including dH_2_O, and ethanol (EtOH) were used to generate CO_2_—NBs. The generation of CO_2_-NBs was accomplished through a novel two-step approach, where initially, CO_2_ gas was bubbled through the solvent at 0.25 Pa for 15 min to achieve pre-saturation, as illustrated in Fig. 1. In the second step, high-pressure nano-jet homogenizer was operated at 80 MPa pressure and the saturated solvent was poured into the sample tube. Solution passed through the interaction chamber at high pressure causes cavitation inside the solvent resulting in nanobubbles with high internal pressure. Three continuous cycles were performed to get the final CO_2_—NBs solution that was immediately tightly sealed in the glass vials to avoid gas leakage and then examined in subsequent tests.

### 2.3 Characterization of CO_2_-NBs

#### 2.3.1 Tyndall effect

The tyndall effect is a well-recognized phenomenon found in colloidal solutions, which serves as the first evidence for the existence of nanobubbles in water-based solutions. Nanobubbles have characteristics feature to scatter the light while passing through it. A green laser light of wavelength 532 ± 10 nm was used to pass through the NBs solution.

#### 2.3.2 Measurement of size and zeta potential

The size of NBs was measured using a method from Javed et al. [11]. Measurements were conducted using a Zeta-sizer Nano ZS (Malvern, United Kingdom), with a scattering angle of 173 degrees and at a temperature of 25 °C. The material's refractive index was adjusted to one.

The zeta potential of NBs was determined using laser doppler velocimetry (LDV) using the Zeta-sizer Nano ZS (Malvern, United Kingdom). The experiment used a sample cell (DTS1070) with a bent capillary. A series of automated runs (1–100) were conducted for each sample, with a time interval of 30 sec between each measurement.

#### 2.3.3 Freeze-thaw of CO_2_-NBs

To validate that the nano entities generated from the CO_2_-NBs solution were really bubbles rather than solid particles, freeze–thaw analysis was conducted using the methodology outlined by Javed et al. [21] with minor modifications, where freezing and subsequent thawing caused the nanobubbles to become unstable and diffuse into the liquid. 20 mL solution of CO_2_-NBs was poured into a glass vial and stored at −18 °C for 12 h. Subsequently, the samples were thawed for a period of 6 h. The size distribution of the NBs was analyzed using dynamic light scattering (DLS) and Zeta-sizer Nano ZS equipment from Malvern.

#### 2.3.4 Effect of solvent type

The effects of two different solvents dH_2_O and EtOH on the generation of CO_2_-NBs were investigated. The two solvents were saturated with CO_2_ gas and passed through the NBs generation method, mean bubble size and zeta potential measurements were done by Malvern Nano ZS.

#### 2.3.5 Effect of CO_2_-saturation time

The first step of CO_2_-NBs involves the saturation of CO_2_ gas in the solvent. Different time periods of 20, 30, and 40 min were evaluated for the generation of CO_2_-NBs in dH_2_O. All these saturated solvents were passed through high-pressure nano-jet homogenizer for the generation of CO_2_-NBs. Malvern Nano ZS was used for the mean bubble size and zeta potential measurements.

#### 2.3.6 Effect of homogenization cycles

During the second phase of CO_2_-NBs production, the saturated solvent is homogenized. A range of homogenization cycles, from one to five, were executed to determine the minimum mean bubble size of the CO_2_—NBs produced. Using DLS analysis, the mean bubble size and zeta potential were characterized.

#### 2.3.7 Stability of CO_2_-NBs

The stability of CO_2_-NBs was evaluated for different storage times. Mean bubble size and zeta potential values were determined at 0, 6, 12, 24, and 48 h of storage by DLS analysis using Malvern Nano ZS.

#### 2.3.8 Transmission electron microscope (TEM) analysis of CO_2_-NBs

The morphological analysis of CO_2_-NBs generated by high-pressure nano-jet homogenization method was conducted using TEM (Hitachi H-7650, Tokyo, Japan). Briefly, the procedure involved preparing samples by immersing a Micro-Electro-Mechanical system (MEMS) copper chip into a suspension of CO_2_-NBs, followed by drying under a yellow lamp for a duration of 3 min, the prepared chip was mounted onto a TEM holder for visualization at high resolution.

### 2.4 Single-Factor analysis for extraction

The single-factor extraction experiment was conducted with the following parameters: ultrasonic power ranging from 200 to 450 W, surfactant concentration ranging from 0.5 to 8 %, and extraction period ranging from 10 to 60 min. The experimental design is shown in Table 1.

**Table 1:** Single-factor experimental design.

Single Factor	Power (W)	Surfactant concentration (%)	Time (min)
Power	200, 250, 300, 350, 400, 450	2	30
Concentration	350	0.5, 1, 2, 4, 8	30
Time	350	2	10,20,30,40,50,60

### 2.5 Response surface design

The three-factor and three-level experimental designs were derived from the single-factor experimental design. The independent factors in the study were ultrasonic power (A), surfactant concentration (B), and extraction time (C), whereas the dependent variable was the extraction yield of total phenolic compounds (TPC). The experiment was enhanced using the Box-Behnken (B-B) design. A total of 20 unique trial combinations were analyzed using Design-Expert 13, with variables and coding levels presented in Table 2.

**Table 2:** Factors and their coding levels in experimental design for response surface methodology (RSM).

Factors	Levels		
	−1	0	1
A: Ultrasonic power (W)	300	350	400
B: Surfactant concentration (%)	2	4	8
C: Ultrasonic time (min)	30	40	50

### 2.6 Determination of TPC

TPC was quantified by the method used by Shirazi et al. [22] with slight modifications. A gallic acid curve was generated by diluting the standard solution of gallic acid in methanol to get concentrations of 10, 20, 40, 60, 80, and 100 µg/mL. A volume of 100 µL from each of these dilutions and samples were combined with 500 µL of water, followed by the addition of 100 µL of Folin-Ciocalteu reagent. The mixture was then left undisturbed for a duration of 6 min. Subsequently, 1 mL of a 7 % sodium carbonate solution and 500 mL of dH_2_O were introduced into the reaction mixture. The spectrophotometric measurement of absorbance was taken at a wavelength of 760 nm after a duration of 30 min.

### 2.7 Extraction methods

#### 2.7.1 Conventional extraction

dH_2_O was used as the solvent for extracting polyphenols. In summary, 4 g of PS was introduced into 20 mL of dH_2_O. The mixture was stirred using a magnetic stirrer for a duration of 40 min at a speed of 200 rpm while being kept in a dark environment. The sample was subjected to centrifugation with a force of 10,000 × g for a duration of 10 min at a temperature of 4 °C in a centrifuge that was kept refrigerated.

#### 2.7.2 Ultrasonic extraction

The extraction of polyphenols from PS was performed using ultrasonic technique, following the approach described by Han et al. [23] with minor modifications. A suspension was prepared by combining 4 g of peanut shell powder (dry weight) with 20 mL of dH_2_O. The extraction was performed using an ultrasonic probe (19 mm) immersed 1 cm into the mixture under dark conditions for 40 min, with operational parameters set at 22.95 kHz frequency and 400 W power output. After ultrasonication, the mixture was immediately centrifuged at 10,000 rpm for 15 min, where the supernatant was collected for phenolic compound analysis while the precipitate underwent freeze-drying for SEM examination.

#### 2.7.3 CO_2_-NBs-assisted ultrasonic extraction

CO_2_-NBs-assisted extraction was performed under the same conditions as described above in Section 2.7.2, CO_2_—NBs solution was used as the extraction solvent.

### 2.8 Characterization of extract

#### 2.8.1 Total phenolic content (TPC)

The TPC of the optimized extract was determined by the method as described in Section 2.6.

#### 2.8.2 Total flavonoid content (TFC)

The quantification of the overall flavonoid concentration was conducted using the aluminum chloride colorimetric technique. 1 mL of PSE sample and 1 mL of a standard quercetin solution (with concentrations of 100, 200, 400, 600, 800, and 1000 µg/mL) were placed into test tubes. Then, 4 mL of dH_2_O and 0.3 mL of a 5 % sodium nitrite solution were added to each tube. 0.3 mL of a 10 % solution of aluminum chloride was added after a duration of 5 min. At the 6th min, a 2 mL solution of 1 M sodium hydroxide was introduced. The final volume was adjusted to 10 mL, resulting in an orange-yellow colored solution, and the absorbance measurements were performed at 510 nm wavelength using a Shimadzu UV-2600 spectrophotometer. The TFC of the extract mixture was quantified as milligrams of quercetin equivalents per 100 g of dry mass.

#### 2.8.3 Antioxidant activity

##### 2.8.3.1 1,1-diphenyl-2-picrylhydrazyl (DPPH)

A DPPH solution of 1.75 × 10^4^ mol/L was prepared with EtOH, 2 mL PSE samples were added to 2 mL DPPH solution and mixed as the sample group. 2 mL samples were added to 2 mL EtOH solution and mixed as the control group. 2 mL EtOH was added to 2 mL DPPH solution and mixed as the blank group, and the reaction time was set to 30 min at room temperature and then the absorption value at 517 nm was detected.

(1) $$Radicalremovalrate\left(\%\right)=\frac{A_{0}-\left(A_{t}-B\right)}{A_{0}}$$

where Ao is the light absorption value of DPPH blank group, A_t_ is the light absorption value of sample group, and B is the light absorption value of control group.

##### 2.8.3.2 Ferric reducing antioxidant power (FRAP) assay

The FRAP test of PSE was conducted using the FRAP assay kit (Beyotime, Beijing, China) in accordance with the manufacturer's instructions. In summary, 180 μL of FRAP working solution and 5 μL of the PSE sample were applied to each well of a 96-well plate. After gentle agitation and 5-min incubation at room temperature, absorbance readings were taken at 595 nm, using deionized water as a positive control. The analysis was performed in triplicate, while a standard curve was generated using FeSO4 solutions of different concentrations.

##### 2.8.3.3 2,2′-Azino-bis (3-ethylbenzothiazoline-6-sulfonic acid) (ABTS) radical scavenging assay

The ABTS radical scavenging activity of PSE was evaluated using ABTS assay kit (Beyotime, Beijing, China) according to the manufacturer protocol. Briefly, ABTS + solution (200 μL) and the sample (10 μL) were added to each well of a 96-well plate. The plate was gently mixed and incubated at room temperature for 5 min, and the absorbance was then measured at 414 nm. The dH_2_O was used as a positive control. All determinations were performed in triplicate.

At the same time, Trolox calibration solutions with a series of concentrations were evaluated to establish a standard curve. Trolox equivalent antioxidant capacity (TEAC) was defined as the concentration of the antioxidant that gives the same percentage reduction in absorbance of ABTS^+^ as 1 μM Trolox does.

#### 2.8.4 High performance liquid chromatography (HPLC)

To analyze the specific components of each phenolic chemical in the extract, the extracts were passed through a filter paper with a pore size of 0.45 µm and then examined using HPLC. The chromatographic separation was performed on a Welch ultimate LP-Column 18 (5 µm, 250 mm × 4.6 mm, China). The mobile phase comprised of solvent (A) containing 0.4 % formic acid and solvent (B) consisting of acetonitrile. The chromatographic settings were as follows: the flow rate was set at 1 mL/min, different concentrations of standard and sample volume of 20 µL were injected, the analysis was conducted at room temperature, and detection was performed at a wavelength of 280 nm. The polyphenols were produced in a standard combination with a concentration of 1 mg/mL. The peaks were determined based on the retention time of the commercially available phenolic compounds.

#### 2.8.5 Scanning electron microscopy (SEM)

To assess the impact of various extraction solutions and circumstances on the surface microstructure of treated peanut shell powder, a SEM (Model SU 5000, Hitachi, Tokyo, Japan) was used to analyze PS samples at high vacuum settings. The magnification conditions were x3.00 k. The PS samples were subjected to freezing and freeze-drying for a duration of 24 h before being analyzed using SEM.

### 2.9 Statistical analysis

The data was reported as the average value plus or minus the standard deviation (SD) of three replications. Statistically significant differences in statistical computations were identified using a one-way analysis of variance (ANOVA) with a significance threshold of 5 %. Furthermore, Duncan’s novel multiple range test was used to assess the disparities in means (*p* < 0.05). The data underwent processing using the statistical analysis software (v 23.0; SPSS; Chicago, IL, USA). The program used for all graph analysis and preparation was Origin Pro 2024.

## 3 Results and discussion

### 3.1 Characterization of CO_2_-NBs

#### 3.1.1 Tyndall effect

**Fig. 2:**
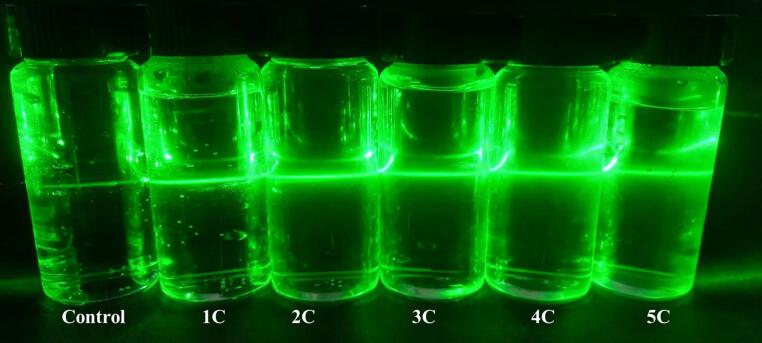
Scattering of the laser beam passing through vials, Tyndall effect of CO_2_-NBs.

The first indication of NBs production is the detection of the tyndall effect. These results were also documented as proof of NBs in other relevant studies [20], [24]. Fig. 2 illustrates a distinct path in the vertical orientation of incoming light in the CO_2_—NBs solution, indicating the presence of colloidal particles in the solution. Given the experimental conditions, it is very probable that the colloid particles are mostly composed of CO_2_—NBs, since the introduction of contaminants is unlikely to occur. The presence of NBs in the solution leads to the scattering of light by the laser beam, which has a wavelength ranging from 495 to 570 nm. The observed scattering indicates the existence of NBs with sizes varying between 100 and 900 nm [25]. This finding was consistent with prior research that documented the presence of NBs in air, nitrogen, oxygen, and carbon dioxide [21], [26].

#### 3.1.2 Size distribution and zeta potential

##### 3.1.2.1 Effect of solvent type

**Fig. 3:**
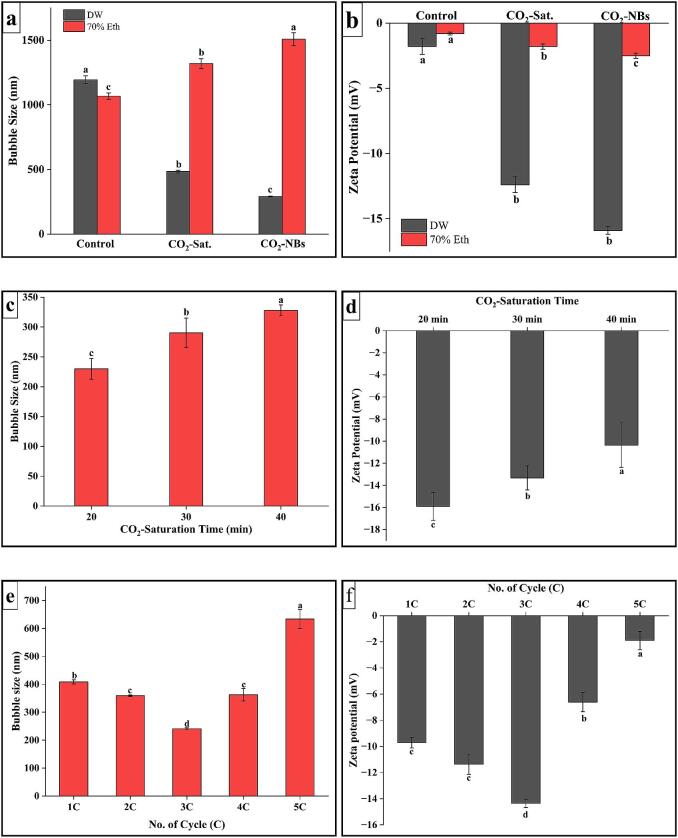

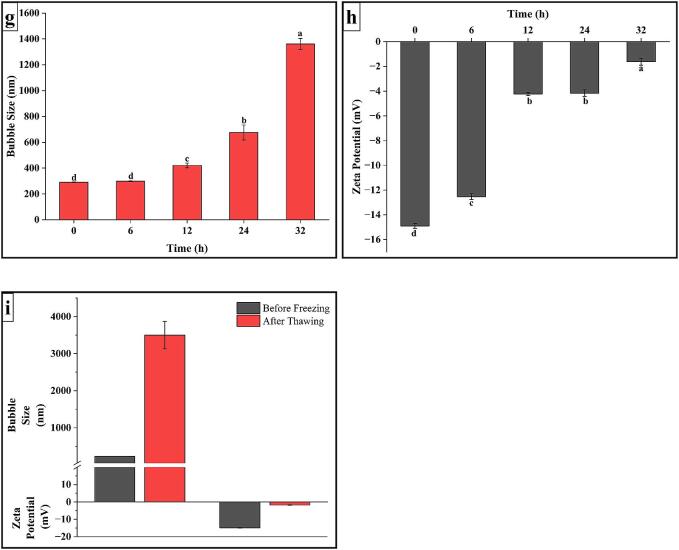
Mean bubble size and zeta potential. (a) Effect of solvent type on mean bubble size, (b) Effect of solvent type on zeta potential, (c) Effect of CO_2_-saturation time on mean bubble size, (d) Effect of CO_2_-saturation time on zeta potential, (e) Effect of homogenization cycles on mean bubble size, (f) Effect of homogenization cycles on zeta potential, (g) Effect of storage time of mean bubble size, (h) Effect of storage time on zeta potential, (i) Effect of freeze–thaw on mean bubble size and zeta potential.

The size of CO_2_—NBs in dH_2_O and 70 % EtOH solution was observed after 15 min of generation, due to the solution becoming transparent, and all microbubbles moved in the solution by buoyancy to gradually disappear. Fig. 3a shows the mean size diameter of NBs generated in two solutions. Mean diameter of CO_2_—NBs in dH_2_O was 250.5 ± 13 nm while DLS measurements of 70 % EtOH CO_2_—NBs showed larger bubble size of 1508 ± 131.5 nm. Nirmalkar et al. [27] elaborated the same findings that nano-tracking analysis (NTA) shows the intensity of nanobubbles decreases with the increase in concentration and no nanobubbles were detected in the mixture containing 70 % EtOH. The primary reason behind having larger bubble size in ethanol solution as compared to water is its physical properties. The viscosity of dH_2_O and EtOH was 0.89 and 1.095 mPa, respectively [28], which significantly affected the nanobubble size, stability, and distribution in both solvents. Higher viscous liquid tends to have larger bubble sizes as the resistance to flow is increased and this may impede the coalescence and diffusion of gas molecules. The particle size analysis depicted in Fig. 3a revealed that both control (without CO_2_ saturation) and CO_2_-saturated samples exhibited significantly larger particle sizes prior to homogenization, confirming the absence of nanobubbles in the initial solutions.

Zeta potential (ZP) is a key indicator of suspended CO_2_-NBs electrical charge in water. As shown in Fig. 3b the negative values of ZP were obtained of all the samples, CO_2_—NBs in dH_2_O having ZP value of −15.9 ± 0.3 mV while for 70 % EtOH solution containing CO_2_-NBs ZP value down to −2.5 ± 0.2 mV. Phan et al. [29] described the same negative ZP values for its samples of CO_2_-NBs in water around − 8.3 ± 0.37  mV. The negative ZP observed in CO_2_-NBs samples may be attributed to the presence of a higher concentration of hydroxyl ions (–OH) at the interface between water and gas. Fig. 3b also elaborated the ZP values of control and CO_2_ saturated group in both solvents which is lower than that of CO_2_-NBs samples indicating the lower bubble stability in solutions.

##### 3.1.2.2 Effect of CO_2_ saturation time

The saturation time of gas is a significant key factor in the generation of nanobubbles as intensity and bubble size are related to dissolved gas in the solvent. Fig. 3c illustrated that minimum bubble size of 229.96 ± 17.44 nm was achieved at 20 min of saturation time. The average size of the bubbles exhibits an upward trend as the duration of CO_2_ saturation rises. Specifically, measurements of 290.4 ± 24.85 nm and 327.93 ± 9.11 nm were recorded at 30 min and 40 min of CO_2_ saturation, respectively. Below 20 min of saturation time, at 10 min, the size of nanobubbles doesn’t have a significant difference but the PDI value increases. Fang et al. [30] explained the same results in which they stated that higher gas saturation leads to larger bubble sizes. According to Meegoda et al. [12], a greater gas flow rate and higher bubble concentrations, even with high zeta potential values, there are chances of bubbles merging to form bigger and unstable microbubbles.

The same trend was observed in measurement of zeta potential values of samples in our study, Fig. 3d showed that the highest zeta value (−15.9 ± 1.27 mV) was reached for CO_2_-NBs samples being saturated for 20 min. Meanwhile, this value decreases with the increase in gas saturation time, as for 30- and 40-min zeta potential values were −13.32 ± 1.09 and −10.36 ± 2.02, respectively. This decrease confirms a reduction in the electrostatic repulsion between the CO_2_-NBs and the surrounding medium, thus increasing the chances of bubble diffusion and increasing the bubble size.

##### 3.1.2.3 Effect of homogenization cycles

dH_2_O was chosen as a solvent to be used for further nanobubble generation. The effect of homogenization cycles on the CO_2_-NBs size was investigated by varying the homogenization cycles from 1 to 5 and the results were presented in Fig. 3e. CO_2_-NBs at different cycles count displays various mean bubble sizes but minimum bubble size was achieved at 3rd cycle. Mean bubble diameter increases with the increase in several cycles *i.e.,* at 1st cycle 408.56 ± 7.25 nm 2nd cycle 359.23 ± 2.82 nm, 3rd cycle 241.16 ± 3.7 nm, 4th cycle 362.9 ± 21.95 nm, and 5th cycle 634 ± 33.78 nm. During the first three cycles of homogenization, the intense mechanical forces effectively break down the macro gas bubbles into nanobubbles. This process is facilitated by the shearing and dispersing action of the homogenizer, which helps to distribute the nanobubbles more uniformly throughout the system. While further increase in homogenization increases the bubble’s size potentially by increasing the temperature, disturbing the viscosity, or accumulating the nanobubbles. Recent studies by Gawin-Mikołajewicz et al. [31] have shown that while increasing homogenization cycles initially reduces particle size and improves uniformity through intense mechanical forces, excessive cycles can lead to reduced efficiency and product quality due to heat generation, which affects surface tension, viscosity, and particle coalescence, ultimately resulting in larger bubble sizes. Further, analysis is needed to explain the mechanism of size distribution during the homogenization process.

Fig. 3f demonstrated the generated CO_2_-NBs at different homogenization cycles (1–5) characterized by varying negative zeta potential (mV) values. The highest zeta value (−14.36 ± 0.32 mV) was recorded at 3rd cycle of homogenization leaving behind the other cycles values that were 1st cycle −9.71 ± 0.41 mV, 2nd cycle −11.36 ± 0.77 mV (second highest charge value), 4th cycle −6.62 ± 0.73 mV, and 5th cycle −1.9 ± 0.7 mV. Obtained charge value can be correlated with the size of the CO_2_-NBs at different homogenization cycles as the dynamics of surface charge distribution. At the 3rd cycle the smallest size bubbles possess a high surface-to-volume ratio, leading to a large concentration of charged ions and molecules at the nanobubble interface. As at different cycles the size increase which leads in decrease of surface-to-volume ratio and the concentration of charged ions and molecules at the nanobubbles interface becomes less pronounce, resulting in lower zeta potential.

##### 3.1.2.4 Stability of the CO_2_-NBs

Fig. 3g indicated that the diameter of CO_2_-NBs samples housed in glass tubes with loosely sealed lids underwent noticeable changes during the time of storage. Specifically, there was an increase in bubble size after storage of 12 h that shifted to 420.8 ± 18.52 nm from the initial size of 237.53 ± 2.9 nm measured after 15 min of NBs generation. During the first 6 h of storage, no significant difference was observed in mean bubble size. However, after 24 h of storage zeta average size was measured at around 675.7 ± 58.13 nm which kept on increasing at 32 h up to size of 1360 ± 43.7 nm. The results were consistent with the findings of Phan et al. [29], wherein a positive association was identified between time of storage and growth of bubble size. According to Wang et al. [26], the increased stability of NBs may be attributed to two factors: the electrical charge of the bubbles at the interface between gas and water, and the high concentration of gas in the medium, which helps prevent the merging of NBs. Evidence has shown that the duration and robustness of CO_2_-NBs often do not exceed 48 h.

Fig. 3h showed that zeta potential of CO_2_-NBs decreased with the increase in storage time. Increasing trend was seen with time as highest value (−14.9 ± 0.19 mV) was observed at 0 h and lowest value (−1.6 ± 0.28 mV) was achieved at 32 h of storage with following trend, 0 < 6 < 12 ≤ 24 < 32 h. The stability of nanobubbles is related to their zeta potential as it generates repulsive forces between the bubbles, reducing their tendency to coalesce or diffuse. Therefore, it can be concluded that with time, the zeta potential value of bubbles tends to approach zero because of diffusion and coalescence.

##### 3.1.2.5 Effect of free-thaw cycle

The average size of CO_2_-NBs was 230.86 ± 2.8 nm before freezing-thaw, and it experienced a substantial and rapid rise to 3499.66 ± 369.06 nm (Fig. 3i). This demonstrated that the vanishing nanosolids were really bubbles, as opposed to droplets or solid particles. Due to its low freezing rate, water causes CO_2_-NBs to come together and merge into larger clusters during the formation of ice crystals. This process is similar to freeze concentration and occurs before the CO_2_-NBs rupture [32]. Therefore, this provided conclusive evidence for the presence of CO_2_-NBs.

Fig. 3i illustrated that before freezing zeta-potential value was −14.9 ± 0.19 mV and changed sharply and reached to −1.8 ± 0.12 mV after free-thaw cycle. Showing the same behavior of disappearance of bubbles cavities and diffusion of gas, thus shifting the gas–water interface charge. This is possibly because of ice crystal formation in liquid that physically disturbs the structure of nanobubbles and directly affects the charge used to be on gas–water interface before freezing. Redistribution of solution after a freeze–thaw cycle may also disturb the surface properties.

##### 3.1.2.6 Morphology of CO_2_-NBs

**Fig. 4:**
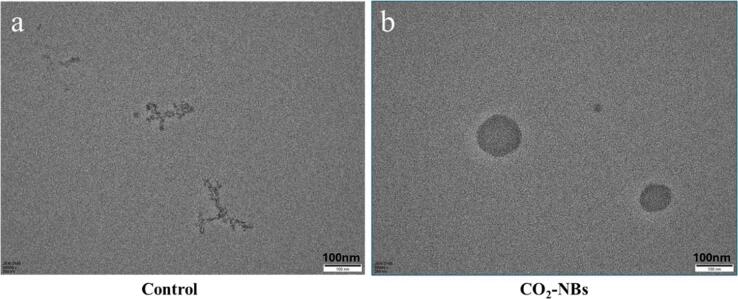
TEM images of distilled water solution, (a) Control group without nanobubbles (NBs) generation, (b) CO_2_-NBs generated with high-pressure nano-jet homogenization method.

The transmission electron microscopy (TEM) pictures depicting nanobubbles were presented in Fig. 4 to enhance the visualization of the nanobubble morphology. CO_2_-NBs were generated with high-pressure nano-jet homogenization method and compared with control group (dH_2_Owithout NBs generation). As a control, Fig. 4a showed the magnified images obtained by TEM at 100 nm with no visible holes or bubbles. Meanwhile, Fig. 4b showed round or oval shaped nonoentities that correspond to characteristics of CO_2_-NBs. This observation underscores the successful generation and stability of CO_2_-NBs. Similar results were obtained by Wang et al. [26] and Javed et al. [21], when they developed NBs with periodic pressure change method and syringe method, respectively. Spherical to oval shaped holes with diameters of 60 to 30 0 nm were observed in the enlarged images of TEM.

### 3.2 Single factor experiment

#### 3.2.1 Effect of solid: Solvent

**Fig. 5:**
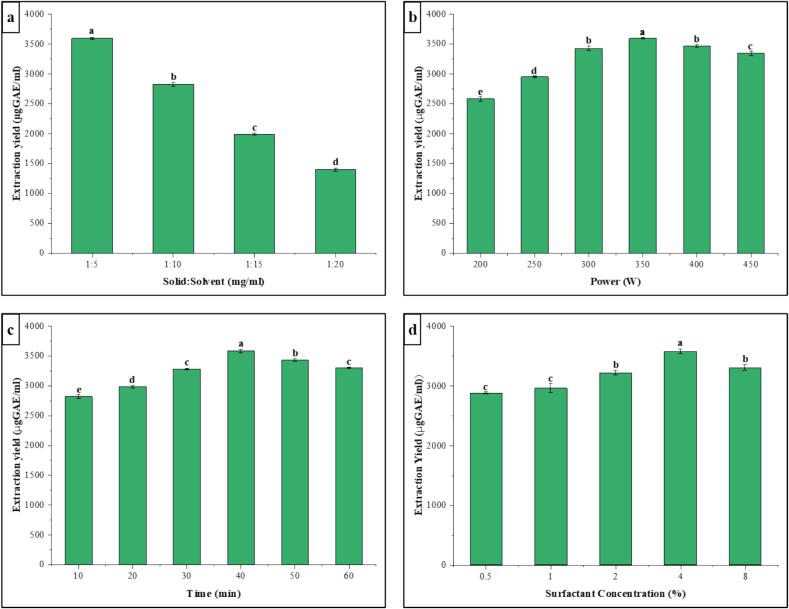
Single factor analysis, (a) Effect of solid: solvent on extraction yield, (b) Effect of ultrasound power on extraction yield, (c) Effect of ultrasound time on extraction yield, (d) Effect of surfactant concentration on extraction yield.

The ideal ratio of solid to solvent in extraction methods is of utmost importance as it directly impacts the efficiency of chemical retrieval, ensures efficient interaction between the solvent and solid structure, and considering practical considerations such as viscosity and equipment limitations. The impact of varying solid-to-solvent ratio (1:5, 1:10, 1:15, and 1:20) on the extraction of polyphenols from PS was examined. Fig. 5a exhibited that highest TPC value of 3595.31 ± 18.61 µg GAE/mL was obtained at 1:5 concentrations, suggesting that this ratio optimized the extraction of phenolic chemicals from the sample matrix. The attempts to utilize reduced solid-to-solvent ratios, specifically below 1:5, led to higher sample thickness, causing practical difficulties for ultrasonic extraction by hindering the ability to sustain effective cavitation and mechanical action. As a result, the extraction of TPC was not optimal. The trend followed by different solvent to solid ratio for highest extraction yield of polyphenols was 1:5 > 1:10 > 1:15 > 1:20 with TPC values 3595.31 ± 18.61, 2825.91 ± 29.89, 1992.4 ± 19.57, and 1398.09 ± 28 µg GAE/mL, respectively. Hence, a 1:5 ratio of solid to solvent was selected for further experimental analysis.

#### 3.2.2 Effect of ultrasonic power

Ultrasonic power significantly improved the extraction yield of TPC as can be seen in Fig. 5b. The yield initially increased with an increase in power magnitude before declining at a higher power value. The extraction yield reached the highest 3597.78 ± 14.89 µg GAE/mL at 350 W. In a study, Xue and Li [33] explained that the yield rose following ultrasonic power treatment because the cavitation action generates shear force, mechanically tears down cell walls, and promotes the migration of compounds. The extraction yield decreased as the ultrasonic power continued to rise after 350 W. Multiple bubbles produced at high power that could interfere with the transmission of ultrasonic waves [34]. Additionally, it could be attributed to the significant high-power effects on the polyphenol structure, the solubility of contaminants, and the damage of active ingredients [35], [36]. Considering the TPC values, 350 W was the ideal power supply for extraction.

#### 3.2.3 Effect of ultrasonic time

The extraction yield has a growing time-dependent effect to a certain time as shown in Fig. 5c. The highest extraction yield of 3582.98 ± 26.67 µg GAE/mL was obtained after 40 min of extraction. Eventually the extraction yield started to decline with prolonged ultrasonication. Initial longer extraction times would expose the sample to ultrasonication completely, leading to cell wall breakage and more intracellular components being released; nevertheless, prolonged ultrasonication would lead to increased compound breakdown due to increased temperature with time [11]. Wang et al. [37] and Sun et al. [38] reported same phenomena in which they found prolonged extraction that reduced the extraction yield. Therefore, 40 min was considered as optimal extraction time.

#### 3.2.4 Effect of surfactant concentration

The impact of surfactant concentration was investigated using a range of concentrations spanning from 0.5 % to 6 %. According to Fig. 5d, it is evident that higher surfactant concentration leads to an increase in TPC. The graph clearly illustrates the positive impact of using 4 % Rhamnolipid, with extraction values reaching 3582.98 ± 39.14 µg GAE/mL. Raising the surfactant concentration to 6 % marginally decreased the extraction efficiency up to 3306.79 ± 49.25 µg GAE/mL. The pattern matched the findings of Javed et al. [11], concluding that 5 CMC rhamnolipid concentration is good for optimal TPC and total flavonoid content (TFC) extraction from *Camellia oleifera* shells. The extraction process is influenced by a wide range of intermolecular interactions. The surfactant structure plays a crucial role in the extraction of phytochemicals by maintaining a delicate balance between hydrophobic and hydrophilic forces. This balance is crucial in facilitating the extraction of the desired components [39]. The optimal yield was determined to be at 4 % rhamnolipid, which was then used in further single-factor tests.

### 3.3 Optimization of extraction process

Given that ultrasonic extraction is the consequence of numerous variables interacting with one another, additional research is needed to determine the impact of various parameters and their interaction on extraction efficiency. Therefore, three parameters (time, solvent concentration, and power) were chosen for response surface methodology (RSM) to further optimize the extraction efficiency with fixed 1:5 solid to solvent ratio.

#### 3.3.1 RSM model fitting

After the single-factor experiment was analyzed, RSM was used to continue with the three-factor and three-level test. In Table 3, 20 experimental combinations were shown. The data were modeled using multiple regression analysis in Design-Expert software. The link between the extraction yield of PSE and the variables was stated by the following equation:

$$Y=3596.26+48.86A1088.69B+52.7C+8.79AB-30.57AC-44.21AB\;-133.46A^{2\;}-189.23B^{2}-124.39C^{2}$$

**Table 3:** Response surface methodology (RSM) Central composite design with coded information.

**Run order**	**A: Power (W)**	**B: Surfactant concentration (%)**	**C: Time (min)**	**Y: Response TPC (µg GAE/mL)**	
				**Experimental**	**RSM**
1	350	4	40	3608.21	3596.26
2	350	0.636414	40	2833.64	2878.24
3	400	6	30	3371.37	3337.60
4	300	6	30	3167.84	3161.17
5	350	4	56.8179	3379.34	3333.06
6	300	6	50	3211.91	3239.27
7	300	2	30	2922.00	2872.94
8	350	4	40	3540.68	3596.26
9	350	4	23.1821	3084.31	3155.81
10	350	4	40	3605.27	3596.26
11	350	4	40	3615.95	3596.26
12	400	2	50	3158.05	3146.90
13	400	2	30	3059.40	3014.21
14	350	7.36359	40	3263.22	3243.83
15	350	4	40	3588.68	3596.26
16	350	4	40	3623.08	3596.26
17	434.09	4	40	3274.52	3300.94
18	265.91	4	40	3137.80	3136.60
19	300	2	50	3111.96	3127.90
20	400	6	50	3262.20	3293.43

Note: Total phenolic content (TPC).

### 3.4 RSM analysis of extraction of TPC

The significance of the fit of the second-order polynomial equation for the data from experiments was estimated using analysis of variance (ANOVA) (Table 4). The substantial results from the quadratic regression model (p < 0.0001) and the absence of fit (p > 0.05) suggest that the mathematical model was dependable for calculating TPC in this investigation.

**Table 4:** RSM analysis of variance (ANOVA) results of experimental model for total phenolic content (TPC).

Source	Sum of Squares	df	Mean Square	F-value	p-value	
Model	1.096E + 06	9	1.218E + 05	54.05	< 0.0001	**significant**
A-Power	32600.15	1	32600.15	14.47	0.0035	
B-Surfactant Concentration	1.613E + 05	1	1.613E + 05	71.60	< 0.0001	
C-Time	37926.21	1	37926.21	16.83	0.0021	
AB	618.29	1	618.29	0.2744	0.6118	
AC	7475.59	1	7475.59	3.32	0.0985	
BC	15638.85	1	15638.85	6.94	0.0250	
A^2^	2.567E + 05	1	2.567E + 05	113.93	< 0.0001	
B^2^	5.160E + 05	1	5.160E + 05	229.02	< 0.0001	
C^2^	2.230E + 05	1	2.230E + 05	98.96	< 0.0001	
Residual	22532.18	10	2253.22			
Lack of Fit	18057.69	5	3611.54	4.04	0.0760	**not significant**
Pure Error	4474.49	5	894.90			
Cor Total	1.119E + 06	19				

**Fig. 6:**
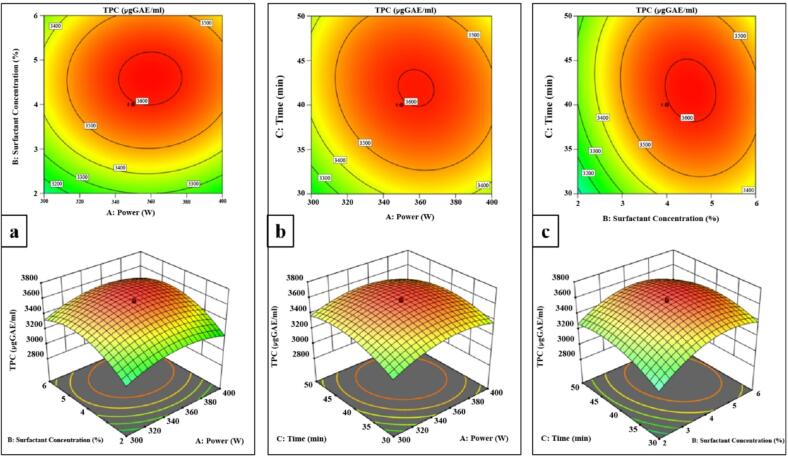
Contour and 3D response surfaces of interactive effects of varied factors, (a) Relationship between ultrasound power and surfactant concentration, (b) Relationship between ultrasound power and ultrasound time, (c) Relationship between surfactant concentration and ultrasound time.

The model’s coefficient of determination (R^2^) was 0.9799, suggesting a prominent level of accuracy. The adjusted determination coefficient (R^2^ adj) was 0.9617, indicating a prominent level of agreement between the observed and anticipated results. The Predicted R2 value of 0.8710 is quite consistent with the Adjusted R2 value of 0.9617, indicating a discrepancy of less than 0.2. Furthermore, the coefficient of variation (CV) was below 10 %, indicating the model's high accuracy and repeatability. To investigate the impact of independent variables on the TPC of PSE, 3D surface plots were generated by altering two variables within the experimental data range while maintaining the third variable constant. The process variables significantly influence the TPC extraction from PSE, as Fig. 6 shows the contour and 3D graphs of RSM findings. At fixed time, the TPC from PSE significantly increased with the elevated surfactant concentration and ultrasonic power up to a certain threshold. Beyond this threshold, further increase in variables reduced the TPC concentration. An optimal point at which the synergetic effects of surfactant concentration and ultrasonic power maximized the TPC was reached (Fig. 6a). Fig. 6b showed the relationship between power and time of ultrasonication for TPC extraction and 3D graph exhibited non-linear relation while achieving an optimal point. However, as ultrasonic power and time increase beyond a certain point, the curve begins to decline, suggesting that there is an optimal point at which maximum TPC is yielded. While Fig. 6c demonstrated the synergetic effect between surfactant concentration and ultrasonic time for optimized TPC values keeping ultrasonic power constant. As variables increased, the TPC followed an upward trend, indicating a positive correlation. However, beyond a certain level, further increase causes reduced TPC values. Our results were in accordance to findings of Javed et al. [11], concluding that initial longer extraction could make the sample completely exposed to ultrasonication, resulting in more release of intracellular components; however, prolonged ultrasonication would cause degradation of compounds due to rise in temperature. Elevated ultrasonic power within the experimental range intensifies cavitation effects, promotes cell wall disruption, and enhances the overall extraction efficiency [40]. However, beyond threshold level, increase in ultrasonic power could potentially result in a Sono-chemical impact, leading to the destruction of intracellular compounds.

### 3.5 Process optimization and verification

Using Design-Expert 13.0′s statistical analysis, the ideal TPC extraction variables have been found to be ultrasonic power 358.76 W, surfactant concentration 4.54 %, extraction duration 41.41 min, and predicted TPC yield was 3619.21 µg GAE/mL. To confirm the experiment’s applicability, the following conditions were used: ultrasonic power 360 W, surfactant concentration 4.5 %, and an extraction time of 41 min. The verification assay yielded 3619.21 ± 113.07 µg GAE/mL (Table 5) with a modest relative standard deviation (RSD) of 0.32 % compared to the model prediction. As a result, the model had good accuracy and consistency. Hence, we were able to extract comparable levels of phenolic compounds using less solvent and in a much shorter time than that reported by Meng et al. [41], Adhikari et al. [42], and Zhang et al. [43].

**Table 5:** Total phenolic content, total flavonoid content, and in vitro antioxidant activities of optimized extract in comparison with conventional and ultrasound method.

Parameters	Conventional extraction	Ultrasonic extraction	CO_2_-NBs assisted ultrasonic extraction
Total phenolic content (TPC) µg GAE/mL	2340.11 ± 80.02^c^	2914.69 ± 145.45^b^	3619.21 ± 113.07^a^
Total flavonoid content (TFC) mg RE/mL	60.3 ± 1.52^c^	77.52 ± 2.13^b^	94.84 ± 4.81^a^
FRAP mM	0.62 ± 0.02^c^	0.93 ± 0.03^b^	1.16 ± 0.07^a^
ABTS mM	4.73 ± 0.09^c^	5.86 ± 0.06^b^	6.78 ± 0.13^a^
DPPH (%)	62.08 ± 0.39^c^	73.97 ± 0.17^b^	81.51 ± 0.33^a^

Note: Mean ± SD (standard deviation) was obtained from three determinations. Different lowercase letters within the same row indicate a significant difference (p < 0.05) among mean homogenous groups of samples with different extraction methods. Ferric reducing antioxidant power (FRAP), 2,2-azino-bis-3-ethylbenzothiazoline-6-sulphonic acid (ABTS), and 2,2-diphenyl-1-picrylhydrazyl (DPPH).

### 3.6 Comparative extraction analysis of PSE by CO_2_-NBs assisted ultrasonic: TFC and antioxidant activity

Quantitative analysis was performed on the PSE to determine TFC, and antioxidant activity using ABTS, FRAP, and DPPH assays extracted by conventional method, ultrasonic method and CO_2_-NBs assisted ultrasonic technique. The examination of several extraction techniques revealed significant disparities in the overall TFC and antioxidant properties of the PSE. Table 5 showed that the TFC was 60.3 ± 1.5 mg RE/g in the traditional extraction method, but the ultrasonic extraction method showed a rise to 77.5 ± 2.1 mg RE/g. Significantly, the use of optimized CO_2_-NBs aided by ultrasonic resulted in a substantial increase in TFC to 94.8 ± 4.8 mg RE/g. This indicates that including CO_2_-NBs into the ultrasound-assisted extraction process improves the extraction efficiency, highlighting the promising potential of this innovative method for maximizing the recovery of flavonoids.

The ABTS test showed a consistent increase in antioxidant activity across the different extraction techniques (Table 5). As we know, Trolox is used as a standard for antioxidant activity, the antioxidant capacity of the sample can be expressed by Trolox-equivalent antioxidant activity (TEAC). The ABTS activity of conventional extraction was measured to be 4.73 ± 0.09 mM, whereas ultrasonic extraction achieved a higher value of 5.86 ± 0.06 mM. The use of CO_2_-NBs in combination with ultrasonic extraction resulted in a significant increase in ABTS activity, reaching a value of 6.78 ± 0.1 mM. Similarly, the FRAP assay results mirrored the trend observed in the ABTS assay. For the FRAP method, total antioxidant activity is expressed as the concentration of FeSO_4_, the standard solution. Table 5 showed that conventional extraction yielded an FRAP activity of 0.621 ± 0.02 mM, ultrasonic extraction increased it to 0.934 ± 0.031 mM, and CO_2_-NBs assisted ultrasonic extraction showed the highest FRAP activity at 1.16 ± 0.007 mM. The observed improvements in both ABTS and FRAP activities suggested that the CO_2_-NBs assisted ultrasonic extraction method enhanced the overall antioxidant potential of the PSE. The DPPH assay results further underscored the efficacy of the extraction methods in capturing the antioxidant potential. Conventional extraction yielded a DPPH activity of 62.08 ± 0.39 %, ultrasonic extraction exhibited an increase to 73.97 ± 0.17 %, and CO_2_-NBs assisted ultrasonic extraction showed the highest DPPH activity at 81.51 ± 0.33 % (Table 5). This trend aligns with the other antioxidant assays, reinforcing the notion that the CO_2_-NBs assisted ultrasonic extraction method enhances the antioxidant capacity of the PSE, potentially due to improved extraction of bioactive compounds.

Our results were comparable to previous studies on the extraction of phytochemicals from PS and the experiment showed that the presented study is cost-effective, uses less solvent, and green extraction technique. In a study, DPPH and ABTS scavenging rate of PSE (water as solvent) were 55.36 ± 0.94 % and 1.51 ± 0.3 %, respectively while the TPC and TFC of PSE (methanol as solvent) values were 3.52 ± 1.54 mg GAE/g and 40.72 ± 5.64 mg RE/g, respectively [41]. Similarly, Adhikari et al. [42] quantify the PSE of different cultivars, using methanol solution in shaking incubator, maximum values of TPC, TFC, DPPH, and ABTS reported for *Akwang* variety were 739.8 ± 7.71 µg GAE/g, 568.0 ± 10.48 µg QE/g, 87.5 ± 1.13 %, and 58.1 ± 1.88 %, respectively. In another study by Zhang et al. [43], it has been reported that PSE obtained by methanolic solution has TPC and TFC values of almost 3.5 mg GAE/g and 75.3 mg RE/g, respectively. Hence, using water as solvent CO_2_-NBs assisted ultrasonic extraction technique can be used for optimized phytochemical extraction.

### 3.7 HPLC-PDA

**Fig. 7:**
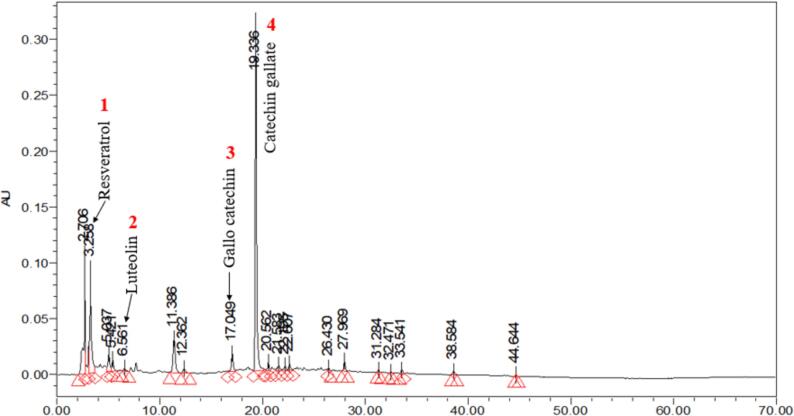
HPLC chromatograph of peanut shell extract (PSE).

Upon conducting an analysis of the PSE using HPLC, it was observed that there were clear peaks that corresponded to different polyphenolic compounds. Standard curves of various polyphenolic compounds were utilized for comparison to identify and measure these compounds. Fig. 7 showed that based on the retention times and spectral characteristics, four compounds −gallocatechin, catechin gallate, resveratrol, and luteolin- were found to be identical. The compound known as resveratrol showed a maximum concentration at a specific period of 3.25 min, whereas luteolin demonstrated a peak later of 6.561 min. Further, the chromatogram showed a prominent peak at 17.04 min, which corresponds to the presence of gallocatechin. Catechin gallate, a polyphenolic component, had a distinct peak at 19.336 min and showed the largest peak area under the curve (AU), suggesting its high concentration in the PSE. The amounts of gallocatechin, catechin gallate, resveratrol, and luteolin were determined using their respective standard curves. Many studies prove that luteolin is the main component of PSE. Zhang et al. [43] reported that luteolin is one of major components in PS extract. In another study by Sun et al. [44], it has been reported that luteolin is the dominant component of PSE. Hence, our study was in accordance with previous findings.

### 3.8 Scanning electron microscopy of peanut shells

**Fig. 8:**
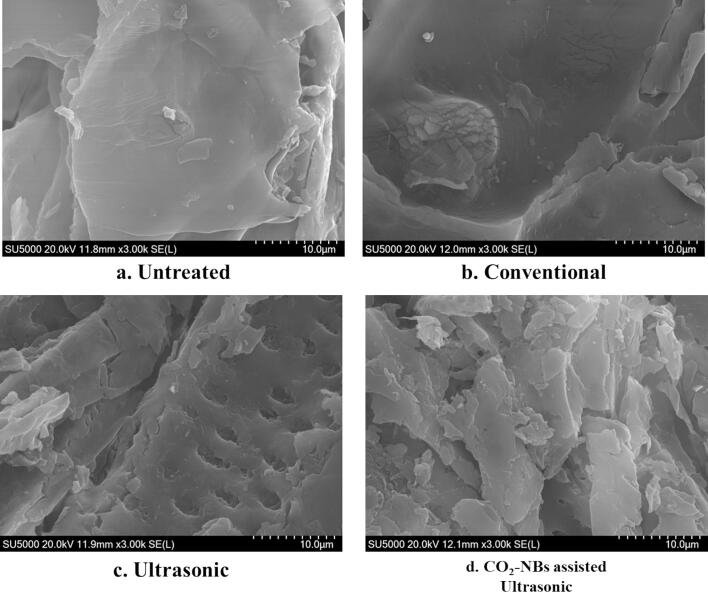
SEM images of peanut shell (PS) after extraction treatments (a) Untreated, (b) Conventional, (c) Ultrasonic, (d) CO_2_-NBs assisted ultrasonic.

SEM was used to analyze the structural changes in PS powder that was procured after three different extraction methods, including CO_2_-NBs assisted ultrasonic extraction, ultrasonic extraction, and traditional extraction with magnetic agitation. In addition, an untreated powder sample was used as a control. Fig. 8 demonstrated very little structural difference in both the untreated powder and the sample that underwent conventional extraction with magnetic stirring. Significant modifications were detected in the samples subjected to the ultrasonic extraction technique as compared to control group. The ultrasonic extraction procedure specifically led to the creation of a porous structure inside the plant shell powder. The presence of a porous morphology indicates that there is some level of disturbance or disintegration of the internal structure of the powder, which might possibly enhance the release of bioactive chemicals. Conversely, the CO_2_-NBs aided ultrasonic extraction approach caused significant damage to the structure of the PS powder as compared to other groups. The powder’s surface exhibited extensive breakage and disruption, clearly showing substantial structural modifications. The observed damage may be ascribed to the synergistic impact of ultrasound and CO_2_-NBs, which can augment the infiltration and disruption of the PS powder. The SEM images showed that the use of CO_2_-NBs assisted ultrasound extraction leads to significant improvements in the extraction efficiency of polyphenolic compounds, likely due to the increased surface area and improved accessibility of bioactive components within the disrupted PS powder.

## 4 Conclusion

The current study investigated the generation of CO_2_-NBs and their application to optimize the green extraction method for the polyphenols from PS. Firstly, CO_2_-NBs were generated using high pressure homogenization method and characterized using DLS and TEM. The results showed the existence of NBs with mean size of 229.96 ± 17.44 nm. Then, the optimization of extraction parameters was performed to extract the polyphenols from PS. The optimized parameters were ultrasonic power 358.76 W, surfactant concentration 4.54 %, and extraction duration 41.41 min. Furthermore, comparative analysis of the optimized extracts confirmed that the CO_2_-NBs assisted ultrasonic technique achieved the highest antioxidant activity, demonstrating its effectiveness for maximizing bioactive compound extraction from agro-waste. In addition, the combined effect of NBs and ultrasonication on structural changes of PS post-extraction was also elaborated.

Thus, a first-of-its-kind high-pressure nano-jet homogenization technique was developed to efficiently generate CO_2_-NBs. This novel green extraction method fully exploits PSE for use as a functional ingredient in the food industry. The developed approach establishes a foundation for future applications in extracting bioactive compounds from various agricultural by-products.

## CRediT authorship contribution statement

**Nayyar Iqbal:** Writing – original draft, Methodology, Investigation, Formal analysis, Conceptualization. **Miral Javed:** Writing – original draft, Data curation. **Ramy M. Khoder:** Writing – original draft, Validation. **Areej Areej:** Writing – original draft, Validation, Methodology. **Renyu Zheng:** Writing – original draft, Investigation, Data curation. **Shanbai Xiong:** Writing – original draft, Visualization, Methodology. **Ibrahim Khalifa:** Writing – review & editing, Writing – original draft, Visualization, Conceptualization. **Hassan Barakat:** Writing – review & editing, Writing – original draft, Supervision, Resources. **Youming Liu:** Writing – review & editing, Writing – original draft, Supervision, Project administration, Funding acquisition.

## Funding

This research was supported by the special Funds for the National Modern Agricultural Industrial Technology System (No. CARS-45).

## Declaration of competing interest

The authors declare that they have no known competing financial interests or personal relationships that could have appeared to influence the work reported in this paper.
